# Synthesis and Herbicidal Activity of New Hydrazide and Hydrazonoyl Derivatives

**DOI:** 10.3390/molecules200814139

**Published:** 2015-08-04

**Authors:** František Šeršeň, Fridrich Gregáň, Matúš Peško, Dana Dvoranová, Katarína Kráľová, Zuzana Matkovičová, Juraj Gregáň, Jana Donovalová

**Affiliations:** 1 Institute of Chemistry, Faculty of Natural Sciences, Comenius University in Bratislava, Mlynská dolina, Bratislava 842 15, Slovakia; E-Mails: kralova@fns.uniba.sk (K.K.); matkovicova@fns.uniba.sk (Z.M.); donovalova@fns.uniba.sk (J.D.); 2 Department of Chemistry, Faculty of Natural Sciences, Matej Bell University, Tajovského 40, Banská Bystrica 974 01, Slovakia; E-Mail: Fridrich.Gregan@umb.sk; 3 Department of Environmental Ecology, Faculty of Natural Sciences, Comenius University in Bratislava, Mlynská dolina, Bratislava 842 15, Slovakia; E-Mail: pesko@fns.uniba.sk; 4 Institute of Physical Chemistry and Chemical Physics, Faculty Chemical and Food Technology, Slovak University of Technology in Bratislava, Radlinského 9, Bratislava 812 37, Slovakia; E-Mail: dana.dvoranova@stuba.sk; 5 Department of Genetics, Faculty of Natural Sciences, Comenius University in Bratislava, Mlynská dolina, Bratislava 842 15, Slovakia; 6 Department of Chromosome Biology, MFPL, University of Vienna, Dr. Bohr-Gasse 7, Vienna 1030, Austria

**Keywords:** green algae, *N*′-[2,6-dinitro-4-(trifluoromethyl)phenyl]hydrazides, *N*′-[2,6-dinitro-4-[trifluoromethyl)phenyl]hydrazonoyl derivatives, photosynthesis inhibition, spinach chloroplasts

## Abstract

Three new hydrazide and five new hydrazonoyl derivatives were synthesized. The chemical structures of these compounds were confirmed by ^1^H-NMR, IR spectroscopy and elemental analysis. The prepared compounds were tested for their activity to inhibit photosynthetic electron transport in spinach chloroplasts and growth of the green algae *Chlorella vulgaris*. IC_50_ values of these compounds varied in wide range, from a strong to no inhibitory effect. EPR spectroscopy showed that the active compounds interfered with intermediates Z^•^/D^•^, which are localized on the donor side of photosystem II. Fluorescence spectroscopy suggested that the mechanism of inhibitory action of the prepared compounds possibly involves interactions with aromatic amino acids present in photosynthetic proteins.

## 1. Introduction

Hydrazides, hydrazonoyl halides and hydrazonoyl cyanides exhibit a wide spectrum of biological activities including antimicrobial [1,2,3,4,5,6,7], antifungal [8,9], antibacterial [8,10], antituberculotic [5,11], anticancer, anti-inflammatory and analgesic effects [12]. The biological activities and applications of hydrazone derivatives were comprehensively reviewed by Rollas and Küçükgüzel [13], Kumar and Narasimhan [14] and Narang *et al.* [15]. Some hydrazine derivatives induced DNA fragmentation [16] and exhibited mutagenic activity [17,18]. Hydrazides of aromatic aldehydes were found to be phytotoxic with specificity for *Amaranthus retroflexus* L. by absorption through the foliage [19]. *N*′-(4-methylphenylsulfonyl)-3-bromothiophene-2-carbohydrazonoyl chloride and 3-(4-benzyl-1-yl)-1-(toluene-4-sulfonyl)-1*H*-thieno[3,2-*c*]pyrazole were synthesized as dopamine D3 receptors [20]. Maleic hydrazide has been used in agriculture as a major commercial herbicide since 1950, however, later studies showed that it exhibits mutagenic effects [21,22].

Hydrazine is an electron donor to the oxidizing side of photosystem II (PSII) and in photosynthesis it supports a light-dependent electron flow in chloroplasts inhibited at the water-oxidizing complex (WOC) [23]. Treatment of thylakoids with hydrazine permits a high population of the redox states S_0_, S_−1_, and S_−2_ in the water oxidase, a complex enzyme which integrates a photochemical reaction centre, PSII, and a catalytic centre, a manganese cluster [24]. According to Förster and Junge [25], two bridging ligands, possible Cl^−^ or OH^−^, which normally connect two Mn nuclei, can be substituted by two molecules of hydrazine when the WOC resides in state S_1_. The reactivity of hydrazine with PSII depends strongly on redox state of WOC [26]. Treatment of chloroplasts with high hydrazine concentration (1 mmol/dm^3^) resulted in complete inhibition of water splitting reaction under flash light [27]. The dichlorophenol-indophenol (DCPIP) reduction by PSII in chloroplasts prepared from leaves of *Phaseolus vulgaris* L. was significantly decreased by maleic hydrazide treatment, while ferricyanide reduction activity was significantly accelerated and it was assumed that the site of action of maleic hydrazide is not situated in PSII but it is between cytochrome and plastocyanin on the donor side of PSI [28]. *N*′-phtalohydrazine and dichlorophenylhydrazine were found to be relatively efficient donors to PSII, although less efficient than hydrazobenzene [29]. The photosynthetic electron transpot (PET) inhibiting activity of hydrazones derived from furo[3,2-*b*]pyrrole-5-carboxhydrazides by their reactions with substituted furan-2-carboxaldehydes or thiophene-2-carboxaldehyde varied in the range from 0.071 to 2.060 mmol/dm^3^ and application of these compounds at concentrations 1–100 μmol/dm^3^ mostly did not affect significantly chlorophyll content in *Chlorella vulgaris* [30]. On the other hand, mixtures containing hydrazone compounds and copper were patented as suitable for controlling the growth of algae [31]. The ionophore carbonylcyanide-4-(trifluoromethoxy)phenylhydrazone acts as an uncoupler of ATP-ase, because it disrupts proton transport-coupled ATP synthesis [32,33].

The synthesis of novel hydrazone derivatives is perspective because of their potential use as antimicrobial or therapeutical compounds. In our current work we prepared three new derivatives of hydrazide and five hydrazonoyl derivatives and analyzed their inhibitory effect on PET in spinach chloroplasts by Hill reaction, fluorescence and EPR spectroscopy.

## 2. Results and Discussion

### 2.1. Chemistry

**Scheme 1:**
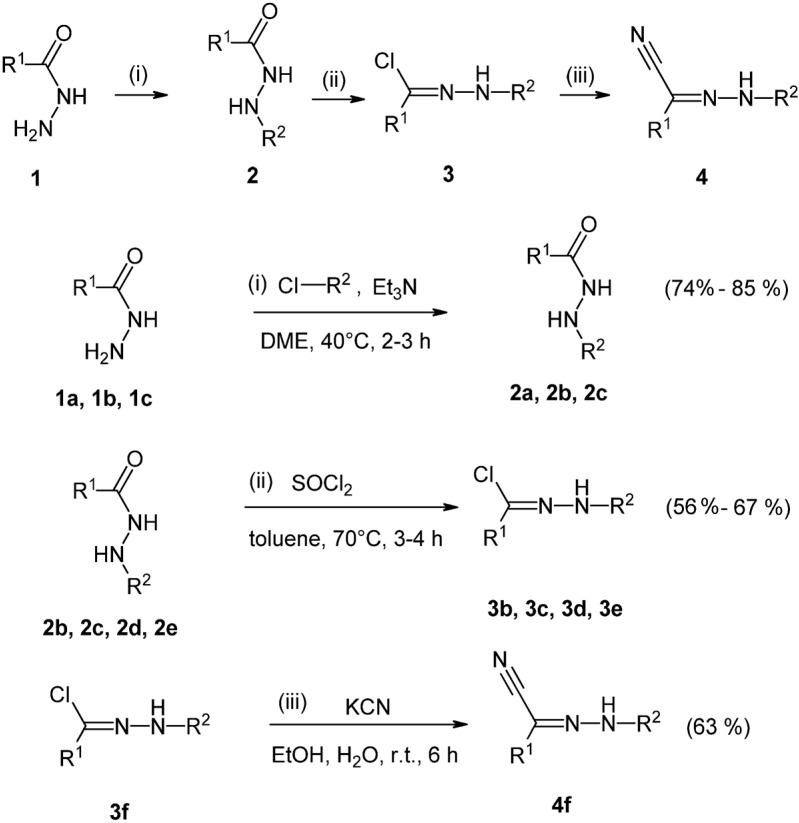
Preparation of *N*′-substituted hydrazides **2** (**2a**, **2b**, **2c**), *N*′-substituted hydrazonoyl chlorides **3** (**3b**, **3c**, **3d**, **3e**) and *N*′-substituted hydrazonoyl cyanide **4** (**4f**); (i), (ii), (iii), reactants and reaction conditions; **1a**, **2a**, R^1^ = 4-methoxyphenyl, **1b**, **2b**, **3b**, R^1^ = 4-*tert*-butylphenyl, **1c**, **2c**, **3c**, R^1^ = methyl; **2d**, **3d**, R^1^ = 4-fluorophenyl, **2e**, **3e**, R^1^ = thiophene-2-yl, **3f**, **4f**, R^1^ = naphtalene-2-yl; R^2^ = 2,6-dinitro-4-(trifluoromethyl)phenyl, for compounds **2**, **3** and **4**. Yields of products (%) are indicated in brackets. Compounds **1a**, **1b** and **1c** were prepared according to literature [34,35,36,37] and compounds **2e**, **2d**, and **3f** were obtained from Tauchem (Bratislava, Slovakia).

The starting compounds for the synthesis of organic hydrazonoyl chlorides **3b**, **3c**, **3d**, **3e** and one hydrazonoyl cyanide **4f**, were carbohydrazides and acethydrazide **1a**–**1c**. These hydrazides were prepared in high yields by nucleophilic substitution reactions of the corresponding methyl esters with hydrazine hydrate in propanol at 80 °C for 10–12 h as described in [34,35,36,37]. *N*-substituted hydrazides **2a**, **2b**, **2c** were prepared by nucleophilic substitution reactions of 1-chloro-4-trifluoromethyl-2,6-dinitrobenzene and the corresponding hydrazides **1** (Scheme 1) in anhydrous dimethoxyethane with triethylamine as catalyst, for 2–3 h at room temperature [38,39,40]. The yields of these products were 74%–80%, after recrystallization.

Hydrazonoyl chlorides **3b**, **3c**, **3d** and **3e** were prepared by nucleophilic substitution reaction of *N*′-substituted hydrazides **2a**, **2b** and **2c** and thionyl chloride [40,41,42,43]. The reaction (Scheme 1) was carried out for 3–4 h in toluene, at 70 °C. The yields of products were 56%–67% after purification. Hydrazonoyl cyanide **4f** was prepared from corresponding hydrazonoyl chloride as starting compound and potassium cyanide in an ethanol–water mixture [40]. The yield was 63% after recrystallization from cyclohexane. All prepared compounds were solid compounds. Hydrazonoyl chlorides and hydrazonoyl cyanide were colored. Structure of all newly prepared compounds was confirmed by ^1^H-NMR, IR spectroscopy and elemental analysis.

### 2.2. Inhibition of Photosynthetic Electron Transport (PET) in Spinach Chloroplasts

Five of the studied compounds inhibited PET in spinach chloroplasts. However, their inhibitory activity varied over a wide range. The IC_50_ values of the studied compounds are presented in Table 1 (second column). The most effective derivative was *N*′-[2,6-dinitro-4-(trifluoromethyl)phenyl]thiophene-2-carbohydrazonoyl chloride (**3e**) with an IC_50_ = 2.34 μmol/dm^3^, which is comparable to the classical herbicide diuron (3-(3,4-dichlorophenyl)-1,1-dimethylurea; DCMU) with an IC_50_ = 1.9 μmol/dm^3^ [44]. The high activity of compound **3e** may be associated with the presence of the thiophene nucleus in this compound because the free electron pair of the sulphur in the thiophene moiety could interact with constituents of the photosynthetic apparatus via hydrogen bonds. On the other hand, no PET inhibition was observed for compounds **2a**, **2c** and **4f**. In our current study, we have chosen 4-trifluoromethyl-2,6-dinitrophenyl substitution for *N*′-substituted hydrazides and hydrazonoyl derivatives based on our previous observations that electron-withdrawing substituents, such as CF_3_, showed effective PET inhibition [45,46,47,48].

**Table 1:** IC_50_ values (μmol/dm^3^) of studied hydrazide and hydrazonoyl derivatives.

Compound	PET Inhibition in Chloroplasts	Growth Inhibition of *Chlorella vulgaris*
**2a**	no inhibition	no inhibition
**2b**	18.0	25.87
**2c**	no inhibition	8.01
**3b**	146.0	17.58
**3c**	2213	10.32
**3d**	61.4	13.32
**3e**	2.34	12.30
**4f**	no inhibition	no inhibition
**DCMU**	1.9 *	7.3 **

Values taken from the literature: *: [44], **: [49].

**Figure 1:**
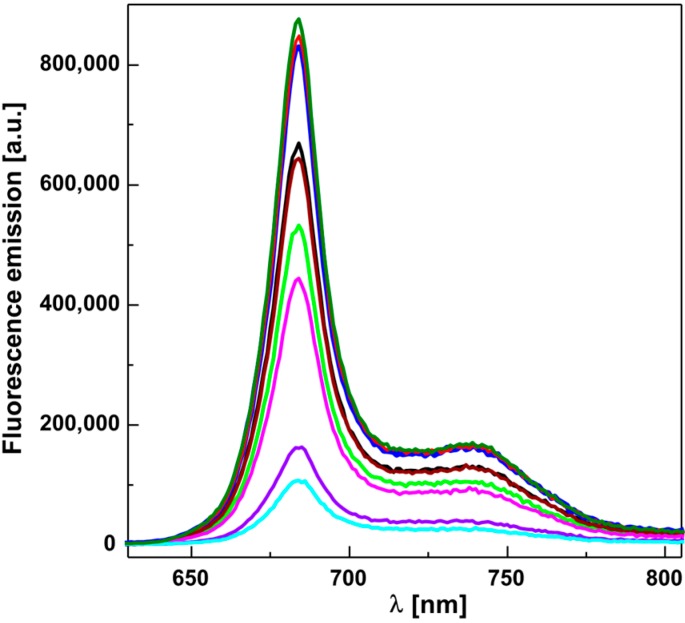
Fluorescence emission spectra of untreated spinach chloroplasts and those treated with 5 μmol/dm^3^ of studied compounds (from top to bottom: **4f**—olive; **2c**—red; **2a**—blue; control sample—black; **3c**—wine; **3e**—green; **3b**—magenta; **3d**—violet; **2b**—cyan).

Next, we attempted to determine the site of action of the studied compounds by studying the emission spectra of chlorophyll in spinach chloroplasts treated with the studied compounds. The fluorescence spectra of spinach chloroplasts treated with compounds under study are presented in Figure 1. We identified the emission peak at 684 nm corresponding to Chl_a_, located in the pigment protein complexes of the photosystem PSII and a shoulder at 739 nm which corresponds to fluorescence of Chl_a_ located in the pigment protein complexes of the PSI [50]. This experiment showed that the compounds which inhibited the PET in spinach chloroplasts quenched the fluorescence of chlorophyll molecules present in the pigment protein complexes of both photosystems. On the other hand, the compounds which did not inhibit PET (**2a**, **2c** and **4f**) increased the fluorescence of chlorophyll. This effect may be caused by defects in photoreduction of Q_A_. A similar effect has been observed by Haveman *et al.* [29] on PSII particles treated with hydrazobenzene. Based on these findings, we can assume that the sites of action of the studied compounds are both photosynthetic centers PSI and PSII.

**Figure 2:**
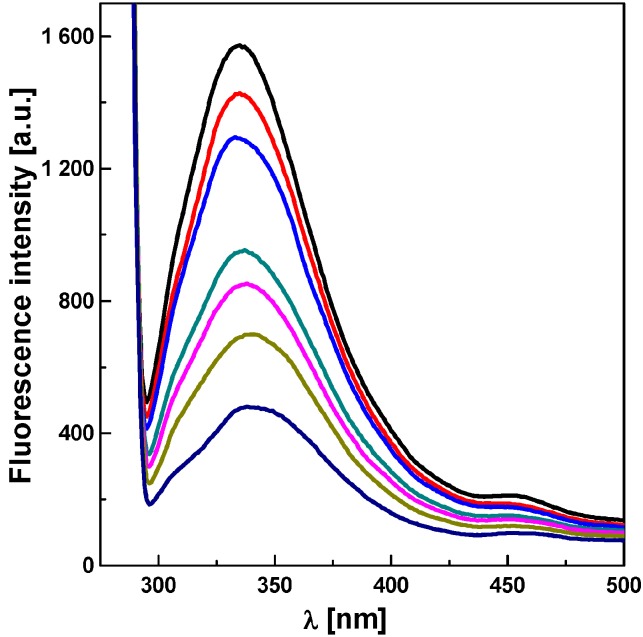
Effect of *N*′-[2,6-dinitro-4-(trifluoromethyl)phenyl]thiophene-2-carbohydrazonoyl chloride on fluorescence of chloroplast amino acids. Compound **3e** was added to spinach chloroplasts to a final concentration of 0, 10, 20, 30, 40, 60, 80 μmol/dm^3^ (from top to bottom).

We speculated that the mechanism of inhibitory action of the studied compounds may involve their interaction with proteins present in the photosynthetic reaction centers. To test our hypothesis, we analyzed the effect of studied compounds on the fluorescence of aromatic amino acids present in spinach chloroplasts. Quenching of the fluorescence was observed when compound **3e** was added to spinach chloroplasts (Figure 2). A similar effect was observed with other tested compounds (data not shown).

**Figure 3:**
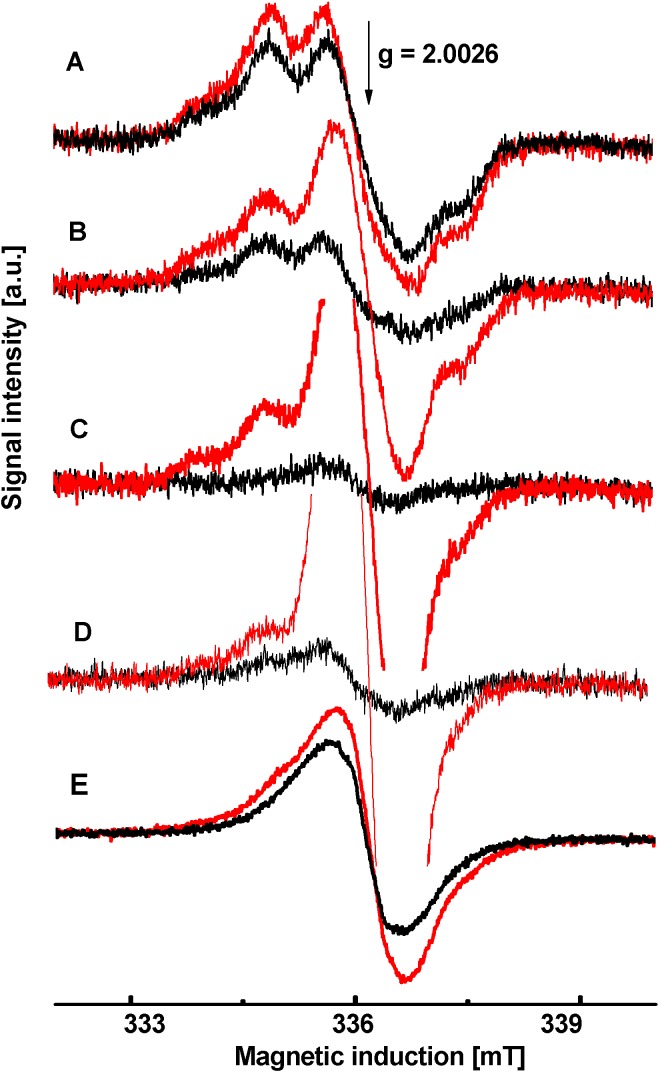
EPR spectra of untreated spinach chloroplasts (**A**) and those treated with 0.05 mol/dm^3^ of **2a** (**B**); **2b** (**C**); **3e** (**D**) and **4f** (**E**). Black curves indicate spectra registered in the dark. Red curves indicate spectra registered in the light.

Using EPR experiments, we found that studied compounds inhibiting the PET immediately decreased the intensity of both EPR signals originating from the intermediates Z^•^ and D^•^, alternatively, only D^•^ (Figure 3). The EPR spectrum of the intermediate D^•^ (g = 2.0046, ∆B_PP_ = 2 mT), which corresponds to the radical of tyrosine at position 161 in the D_2_ protein of PSII [51], is shown as black line in the EPR spectrum of untreated chloroplasts in the dark (Figure 3A, black line). The increase of intensity in the EPR spectra of untreated chloroplasts in the light represents EPR signal of the intermediate Z^•^ (Figure 3A, difference between black and red lines; g = 2.0046, ∆B_PP_ = 2 mT), which corresponds to the radical of tyrosine situated at position 161 in the D_1_ protein of PSII [52]. We found that the studied compounds had different impacts on the intermediates Z^•^/D^•^. Substances that showed no inhibition of the Hill reaction (**2a** and **2c**) exhibited very little effect on intermediates Z^•^/D^•^ which resulted in only minor changes in the EPR spectra of chloroplasts treated with these compounds. EPR spectra of chloroplasts treated with compound **2a** are shown in the Figure 3B. We observed a similar effect in chloroplasts treated with compound **2c** (data not shown). Compounds that inhibited the Hill reaction affected differently function of intermediates Z^•^/D^•^. Almost complete disappearance of the signal corresponding to D^•^ was observed in EPR spectra of chloroplasts treated with compounds **2b**, **3b**, **3c** and **3d** (Figure 3C, black line), whereas the signal from the intermediate Z^•^ remained unchanged (Figure 3C red line). EPR spectra of chloroplasts treated with compound **2b** are shown in the Figure 3C. We observed a similar effect in chloroplasts treated with compound **3b**, **3c** and **3d** (data not shown). On the other hand, compounds **3e** and **4f** interfered with both intermediates Z^•^/D^•^ which is demonstrated by the complete disappearance of both signals in the EPR spectra of chloroplasts treated with these agents (Figure 3D,E, black and red lines). One possible explanation is that the interaction of studied compounds with intermediates Z^•^/D^•^ resulted in interruption of electron transfer from PSII to PSI that caused a large increase of the EPR signal (g = 2.0026, ∆B_PP_ = 1 mT) corresponding to the core of PSI (P680^+^), thus oxidized form of Chl_a_ dimer [53] (Figure 3C,D, red lines). The compound **4f** oxidized P680 already in the dark (Figure 3E, black line).

Interestingly, in chloroplasts in which PET was inhibited by addition of active compounds (*i.e.*, **2b**, **3b**, **3c**, **3d** and **3e**), a recovery of the PET nearly to the original levels was observed after the addition of DPC. This experiment suggests that the studied compounds may act at the donor side of PSII in the complex decomposing water or at the sites of Z^•^/D^•^ intermediates. Our finding is consistent with previous findings of Heath [23] and Förster and Junge [25]. In order to determine whether PET through PSI is damaged, we carried out experiments with chloroplasts treated with the studied compounds and DCPIPH_2_, an artificial electron donor operating in plastocyanin on the donor side of PSI and using methyl viologen as the final artificial electron acceptor of PSI. We found that in such treated chloroplasts, with the exception of the compound **4f** (which caused oxidation of P680 already in the dark), PET through PSI was not interrupted. Thus, it is likely the studied compounds have other site of action, for example, cytochrome *b*_6_*f* complex, located between the PSI and PSII. Interestingly, a similar conclusion was published by Haveman *et al.* [29], who found that hydrazobenzene, which is structurally similar to compounds synthesized in our current study, can be oxidized in at least two photoreactions. In the first reaction, it acts as an efficient donor for PSII and this reaction is inhibited by 5 μM DCMU, while a second hydrazobenzene-DCPIP reaction, which is not inhibited by DCMU, is presumably catalyzed via an oxidized component of the redox chain between the primary stable electron acceptor of PSII and the quencher of chlorophyll fluorescence and PSI.

### 2.3. Inhibition of Algae Growth

To determine a long-time effect of the studied compounds on a photosynthetic organism, we analyzed the effect of these compounds on growth of the green algae *Chlorella vulgaris* monitoring chlorophyll concentrations in algal suspensions according to Pavlíková *et al.* [54]. Six compounds effectively inhibited the growth of algae (Table 1, third column). For an illustration, the antialgal activity of DCMU is about 7.3 μmol/dm^3^ [49]. Interestingly, the most effective compound was **2c**, which did not inhibit PET in spinach chloroplasts. This suggests that the compound **2c** acts in algae on targets other than photosynthetic centers, for example, it may inhibit enzymes involved in biosynthesis of chlorophyll or stimulate some other enzymes involved in its degradation.

## 3. Experimental Section

### 3.1. General Information

Ethyl acetate p.a., *n*-hexane p.a., propanol p.a., ethanol p.a., 2,6-dichlorophenol–indophenol (DCPIP), 1,5-diphenylcarbazide (DPC), TRIS, MgCl_2_, saccharose, 1,1′-dimethyl-4,4′-bipyridinium dichloride hydrate (methyl viologen), dimethoxyethane (DME), triethylamine, dimethylsulfoxide p.a. (DMSO), 3-(3,4-dichlorophenyl)-1,1-dimethylurea (DCMU) and thionyl chloride were purchased from Centralchem (Bratislava, Slovakia). 1-Chloro-4-trifluoromethyl-2,6-dinitrobenzene was purchased from Alfa Aesar (Ward Hill, MA, USA). 4-Ethoxybenzohydrazide, acethydrazide and 4-*tert*-butylbenzohydrazide were prepared according to literature [34,35,36,37]. *N*′-[2,6-Dinitro-4-(trifluoromethyl)phenyl]thiophene-2-carbohydrazide (**2e**), *N*′-[2,6-dinitro-4-(trifluoromethyl)phenyl]-4-fluorobenzohydrazide (**2d**) and *N*′-[2,6-dinitro-4-(trifluoromethyl)]-2-naphthohydrazonoyl chloride (**3f**) were purchased from Tauchem (Bratislava, Slovakia).

Melting points were determined on a Kofler hot plate apparatus and are uncorrected. IR spectra were obtained on a NICOLET NEXUS 470 spectrophotometer in KBr. Elemental analyses were obtained on Elemental Analyzer Carlo Erba CHNS-OEA 1108. ^1^H-NMR spectra at 300 MHz were obtained on Varian Gemini 2000 spectrophotometer in DMSO-*d*_6_ with tetramethylsilane as an internal standard. The purity of prepared compounds and course of reactions were checked on Merck TLC Silica gel 60 F_254_ plates in ethyl acetate–*n*-hexane as the mobile phase.

### 3.2. Synthesis

#### 3.2.1. General Procedure for Synthesis of *N*′-[2,6-dinitro-4-(trifluoromethyl)phenyl]hydrazides **2a**, **2b**, **2c**

A solution of 1-chloro-2,6-dinitro-4-(trifluoromethyl)benzene (10 mmol) in 1,2-dimethoxyethane (20 mL) was added dropwise to a stirred solution of of *N*′-substituted hydrazide **1a**, **1b**, **1c** (10 mmol) and triethylamine (10 mmol) in anhydrous dimethoxyethane (40 mL) over a period of 30 min at 20–25 °C. Then reaction mixture was heated to 40 °C and stirred for 2–3 h. The reaction was monitored by TLC. The reaction mixture was poured on ice water (200 mL). The obtained solid product was filtered off, washed with water and recrystallized from 80% acetic acid.

##### N′-[2,6-Dinitro-4-(trifluoromethyl)phenyl]-4-methoxybenzohydrazide (**2a**)


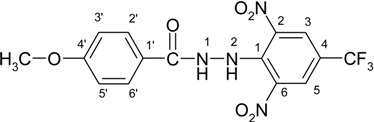

Yield 85%; yellow solid, Mp 188–190 °C (80% AcOH). Anal. Calcd. for C_15_H_11_F_3_N_4_O_6_ (400.28) C, 45.01; H, 2.77: N, 14.00. Found: C, 44.92; H, 2.70; N, 13.84%. IR: 3305 (v/NH), 1674, 1637(v/C=O), 1531, 1536 (v/NO_2_). ^1^H-NMR δ: 10.75 (s, 1H, NH-2), 9.85 (s, 1H, NH-1), 8.53 (q, 2H, *J*_(H-3,CF3)_ = 0.5 Hz, H-3, H-5), 7.74–7.71 (m, AA′XX′, 2H, *J*_(AX)_ = 8.9 Hz, H-2′, H-6′), 7.06–7.03 (m, AA′XX′, 2H, *J*_(AX)_ = 8.9 Hz, H-3′, H-5′), 3.83 (s, 3H, CH_3_).

##### 4-(tert-Butyl)-N′-[2,6-dinitro-4-(trifluoromethyl)phenyl]benzohydrazide (**2b**)


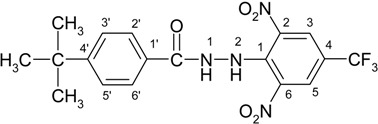

Yield 74%; yellow solid, Mp 214–215 °C (80% AcOH). Anal. Calcd. for C_18_H_17_F_3_N_4_O_5_ (426.35) C, 50.71; H, 4.02: N, 13.37. Found: C, 50.66; H, 3.90; N, 13.24%. IR: 3313 (v/NH), 1655 (v/C=O), 1505, 1542 (v/NO_2_). ^1^H-NMR δ: 10.82 (s, 1H, NH-2), 9.89 (s, 1H, NH-1), 8.54 (br, s, 2H, H-3, H-5), 7.70–7.67 (m, AA′XX′, 2H, *J*_(AX)_ = 8.6 Hz, H-2′, H-6′), 7.54–7.51 (m, AA′XX′, 2H, *J*_(AX)_ = 8.6 Hz, H-3′, H-5′), 1.31 (s, 9H, C(CH_3_)_3_).

##### N′-[2,6-Dinitro-4-(trifluoromethyl)phenyl]acethydrazide (**2c**)


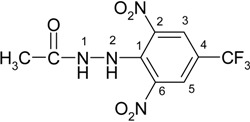

Yield 80%; yellow solid, Mp 234–235 °C (80% AcOH). Anal. Calcd. for C_9_H_7_F_3_N_4_O_5_ (308.18) C, 35.08; H, 2.29: N, 18.18. Found: C, 36.19; H, 2.16; N, 18.04%. IR: 3236, 3327 (v/NH), 1674(v/C=O), 1534, 1577 (v/NO_2_). ^1^H-NMR δ: 10.26 (s, 1H, NH-2), 9.66 (s, 1H, NH-1), 8.52 (q, 2H, *J*_(H-3,CF3)_ = 0.7 Hz, H-3, H-5), 1.76 (s, 3H, CH_3_).

#### 3.2.2. General Procedure for Synthesis of *N*′-[2,6-dinitro-4-(trifluoromethyl)phenyl]hydrazonoyl Chlorides **3b**, **3c**, **3d**, **3e**

Thionyl chloride (7 mmol) was added portionwise over a period of 20 min to a stirred solution (or suspension) of *N*′-[2,6-dinitro 4-(trifluoromethyl)phenyl] hydrazides (6 mmol) **2a**, **2b**, **2c**, in toluene (30 mL). Then reaction mixture was heated to 70 °C over 3–4 h. The reaction was monitored by TLC. The mixture was washed with ice water, 5% aqueous solution of sodium bicarbonate (3 × 10 mL) and then with water. Organic layer was dried with anhydrous Na_2_SO_4_. The mixture was filtered and toluene was evaporated from the filtrate by distillation. The crude product was purified by recrystallization.

##### 4-(tert-Butyl)-N′-[2,6-dinitro-4-(trifluoromethyl)phenyl]benzohydrazonoyl chloride (**3b**)


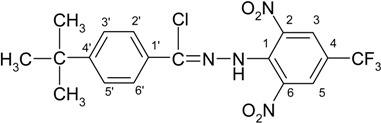

Yield 56%; yellow solid, Mp 201–203 °C (cyclohexane). Anal. Calcd. for C_18_H_16_ClF_3_N_4_O_4_ (444.80) C, 48.61; H, 3.63; N, 12.60. Found: C, 48.65; H, 3.48; N, 12.69%. IR: 3427 (v/NH), 1635(v/C=O), 1570, 1571 (v/NO_2_). ^1^H-NMR δ: 11.35 (s, 1H, NH), 8.73 (q, 2H, *J*_(H-3,CF3)_ = 0.6 Hz, H-3, H-5), 7.74–7.69 (m, AA′XX′, 2H, *J*_(AX)_ = 8.8 Hz, H-2′, H-6′), 7.60–7.56 (m, AA′XX′, 2H, *J*_(AX)_ = 8.8 Hz, H-3′, H-5′), 1.31 (s, 9H, C(CH_3_)_3_).

##### N′-[2,6-Dinitro-4-(trifluoromethyl)phenyl]acethydrazonoyl chloride (**3c**)


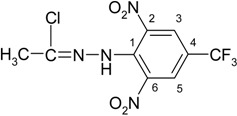

Yield 67%; yellow solid, Mp 97–98 °C (cyclohexane). Anal. Calcd. for C_9_H_6_ClF_3_N_4_O_4_ (326.63) C, 33.10; H, 1.85; N, 17.15. Found: C, 33.28; H, 1.68; N, 17.27%. IR: 3244, 3326 (v/NH), 1539, 1573 (v/NO_2_). ^1^H-NMR δ: 10.96 (s, 1H, NH), 8.66 (q, 2H, *J*_(H-3,CF3)_ = 0.5 Hz, H-3, H-5), 2.38 (s, 3H, CH_3_).

##### N′-[2,6-Dinitro-4-(trifluoromethyl)phenyl]-4-fluorobenzohydrazonoyl chloride (**3d**)


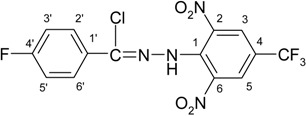

Yield 63%; yellow solid, Mp 181–183 °C (cyclohexane). Anal. Calc. for C_14_H_7_ClF_4_N_4_O_4_ (406.69) C, 41.35; H, 1.73; N, 13.78. Found: C, 41.44; H, 1.88; N, 13.79%. IR: 3242(v/NH), 1634(v/C=N), 1534, 1577 (v/NO_2_). ^1^H-NMR δ: 11.33 (s, 1H, NH), 8.73 (q, 2H, *J*_(H-3,CF3)_ = 0.5 Hz, H-3, H-5), 7.85–7.78 (m, 2H, H-2′, H-6′), 7.47–7.39 (m, 2H, H-3′, H-5′).

##### N′-[2,6-Dinitro-4-(trifluoromethyl)phenyl]thiophene-2-carbohydrazonoyl chloride (**3e**)


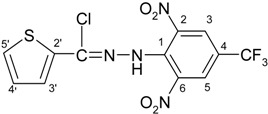

Yield 65%; orange solid, Mp 188–189 °C (cyclohexane). Anal. Calc. for C_12_H_6_ClF_3_N_4_O_4_S (394.71) C, 36.52; H, 1.53; N, 14.19. Found: C, 36.62; H, 1.50; N, 13.39%. IR: 3237(v/NH), 1633 (v/C=O), 1537, 1580 (v/NO_2_). ^1^H-NMR δ: 11.22 (s, 1H, NH), 8.70 (br, s, 2H, H-3, H-5), 7.87 (dd, 1H, *J* = 1.2 Hz, *J* = 5.0 Hz, H-5′), 7.63 (dd, 1H, *J* = 1.2 Hz, *J* = 3.8 Hz, H-3′), 7.20 (dd, 1H, *J* = 3.8 Hz, *J* = 5.0 Hz, H-4′).

##### N′-[2,6-Dinitro-4-(trifluoromethyl)phenyl]-2-naphtohydrazonoyl cyanide (**4f**)


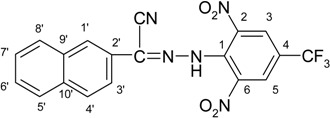


Solid *N*′-[2,6-dinitro-4-(trifluoromethyl)phenyl]-2-naphtohydrazonoyl chloride was added portionwise over a period of 30 min at room temperature to a solution of potassium cyanide (0.33 g, 5 mmol) in ethanol (20 mL) and water (14 mL). After that ethanol (10 mL) and water (7 mL) were added into the reaction mixture and stirred at room temperature for 12 h. The reaction was monitored by TLC. The reaction mixture was poured into water (30 mL). The crude solid product was filtered, washed with water and vacuum-dried. The crude product was recrystallized from cyclohexane. Yield 1.08 g (63%); yellow solid, Mp 253–255 °C (cyclohexane). Anal. Calc. or C_19_H_10_F_3_N_5_O_4_ (429.32) C, 53.16; H, 2.35; N, 16.31. Found: C, 52.98; H, 2.46; N, 16.50%, IR 3232 (v/NH), 2216 (v/CN), 1525, 1537 (v/NO_2_), 1634 (v/CN). ^1^H-NMR δ: 11.88 (br, s, 1H, NH), 8.74 (br, s, 2H, H-3, H-5), 8.26 (br, s, 1H, H-1′), 8.14–7.98 (m, 3H, H-4′, H-5′, H-8′), 7.71–7.63 (m, 3H, H-3′, H-6′, H-7′).

### 3.3. PET Study

PET was monitored in spinach chloroplasts prepared according to our previous work [55]. PET through PSII was monitored by the Hill reaction with DCPIP as an artificial electron acceptor, or by using DPC as an electron donor for intermediate D in PSII [56]. PET through PSI was monitored using DCPIPH_2_ as an electron donor and methyl viologen as an artificial electron acceptor for PSI [56]. DCPIP photoreduction or oxidation of DCPIPH_2_ was determined spectrophotometrically (Genesys 6, Thermo Scientific, Waltham, MA, USA). The chlorophyll (Chl) concentration in these experiments was 30 mg/dm^3^. The inhibitory activities of the studied compounds were expressed by IC_50_ values, *i.e.*, molar concentrations of the compounds causing 50% decrease of absorbance at 600 nm compared to control sample. The effect of the studied compounds on the growth of the green algae *Chlorella vulgaris* was analyzed as described in our previous work [54].

Chlorophyll fluorescence of spinach chloroplasts was recorded at room temperature by spectrofluorimeter FSP 920 (Edinburgh Instruments, Livingston, UK) using an excitation wavelength λ_ex_ = 436 nm. Fluorescence of aromatic amino acids was monitored by a F-2000 spectrophotometer (Hitachi, Tokyo, Japan) using excitation wavelength λ_ex_ = 275 nm, according to our previous work [57]. Both fluorescence experiments were performed in 1 cm fluorescence cell in the right-angle arrangement. The chlorophyll concentration in chloroplast suspension was 10 mg/dm^3^.

EPR experiments were performed by the X-band EPR spectrometer (EMX Plus, Bruker, Germany) at 5 mW microwave power and 0.5 mT modulation amplitude. The chloroplast suspensions were mixed with studied compounds directly before the EPR measurements and immediately transferred to a small quartz flat cell (WG 808-Q, optical cell length 0.04 cm; Wilmad-LabGlass, Vineland, NJ, USA). The samples were irradiated at 295 K directly in the EPR resonator through 5 cm water filter, and the EPR spectra were recorded *in situ* during continuous photoexcitation. The irradiation source was a 150 W halogenated lamp. The chlorophyll concentration was 4.0 g/dm^3^.

Due to the limited solubility of the samples, these were added to the chloroplast suspensions as a DMSO solution. DMSO at a concentration of 10% did not influence the above-mentioned photochemical reaction in the chloroplast (data not shown).

## 4. Conclusions

In this work we prepared three new *N*′-[2,6-dinitro-4-(trifluoromethyl) phenyl)]hydrazide derivatives and five *N*′-[2,6-dinitro-4-(trifluoromethyl)phenyl)]hydrazonoyl derivatives. Hydrazides were synthesized by nucleophilic substitution reactions of the corresponding methyl esters with hydrazine hydrate. Four hydrazonoyl chlorides were prepared by reaction of the corresponding *N*′-substituted hydrazides with thionyl chloride. Starting compounds for the preparation of *N*′-[2,6-dinitro-4-(trifluoromethyl)-phenyl]-2-naphthohydrazonoyl cyanide were *N*′-[2,6-dinitrophenyl-4-(trifluoromethyl)]-2-naphthohydrazonoyl chloride and potassium cyanide. The chemical structures of these compounds were confirmed by ^1^H-NMR, IR spectroscopy and elemental analysis. The majority of compounds exhibited inhibitory effect on photosynthesis in spinach chloroplasts and on growth of the green algae *Chlorella vulgaris*. The IC_50_ values of these compounds varied in wide range, from a strong to no inhibitory effect. EPR spectroscopy showed that the active compounds interfered with intermediates Z^•^/D^•^, which are localized on the donor side of PSII. Fluorescence spectroscopy suggested that the mechanism of inhibitory action of the prepared compounds possibly involves interactions with aromatic amino acids present in photosynthetic proteins.
